# Personal

**Published:** 1911-06

**Authors:** 


### PERSONAL

Dr. Frederic S. Mason read a paper on the Lactic acid ester of santalol and santalol compounds at the meeting of the New York section of the American Chemical Society at the Chemists' Club on May 5.

Dr. Eugene H. Porter, New York State commissioner of health, visited Buffalo on May 12, for the purpose of inspecting the Buffalo State Hospital.

Dr. James W. Putnam, of Buffalo, attended the meeting of the American Neurological Association at Baltimore, May 10 and 12.

Dr. A. B. Allen has removed from No. 868 Main street to No. 20 Allen street, Buffalo.

Dr. Alfred E. Diehl has removed to No. 448 Delaware avenue, Buffalo.

The engagement is announced of Dr. Frederick M. Lemen of Buffalo, and Miss Lauretta S. Kurtz, the wedding to take place in July.

Dr. D. H. McCoy has been elected vice-president and Dr. N. W. Bodenbender, a director of the Clef Club of Buffalo.

A public meeting under the auspices of the Erie County Medical Society and the Buffalo Academy of Medicine was held at the

Central Y. M. C. A. auditorium on Monday evening, May 15, at which Dr. J. N. McCormack, chairman of the organisation committee of the American Medical Association, spoke on "Things about doctors which doctors and other people ought to know." Mayor Fuhrmann, of Buffalo, presided and addresses were delivered by the president of the Chamber of Commerce; Hon. H. P. Bissell, of the New York State Lunacy Commission and Rev. F. T. Parr, rector of St. Mary's Catholic Church. On Tuesday, May 16, Dr. McCormack gave a special "Heart to heart talk" to the medical profession.

---

Dr. Maud J. Frye has removed her office and residence from 224 Allen Street, to 211 Highland Avenue. Hours: 9 A. M., 2 to 3.30 P. M., evenings and Sundays by appointment only. Telephones: Bryant 315; Frontier 38284.

---

The beautiful tribute to the memory of the late Dr. William Warren Potter, adopted by the New York State Board of Medical Examiners, was prepared by the secretary of the board, Dr. Maurice J. Lewi, of New York.

---

Dr. and Mrs. W. G. Grove, of Buffalo, sailed on the Lusitania, May 31st for a three months' tour abroad. They will visit France, Germany, England and other European countries. Dr. Grove will spend a part of the time studying the methods used in the larger foreign hospitals.

